# Genomic variation in the genus *Beta* based on 656 sequenced beet genomes

**DOI:** 10.1038/s41598-023-35691-7

**Published:** 2023-05-27

**Authors:** Sabine Felkel, Juliane C. Dohm, Heinz Himmelbauer

**Affiliations:** Institute of Computational Biology, Department of Biotechnology, University of Natural Resources and Life Sciences, Muthgasse 18, 1190 Vienna, Austria

**Keywords:** Computational biology and bioinformatics, Genome informatics, Plant sciences, Natural variation in plants, Plant domestication, Plant genetics

## Abstract

Cultivated beets (*Beta vulgaris* ssp. *vulgaris*) constitute important crop plants, in particular sugar beet as an indispensable source of sucrose. Several species of wild beets of the genus *Beta* with distribution along the European Atlantic coast, Macaronesia, and throughout the Mediterranean area exist. Thorough characterization of beet genomes is required for straightforward access to genes promoting genetic resistance against biotic and abiotic stress. Analysing short-read data of 656 sequenced beet genomes, we identified 10 million variant positions in comparison to the sugar beet reference genome RefBeet-1.2. The main groups of species and subspecies were distinguishable based on shared variation, and the separation of sea beets (*Beta vulgaris* ssp. *maritima*) into a Mediterranean and an Atlantic subgroup as suggested by previous studies could be confirmed. Complementary approaches of variant-based clustering were employed based on PCA, genotype likelihoods, tree calculations, and admixture analysis. Outliers suggested the occurrence of inter(sub)specific hybridisation, independently confirmed by different analyses. Screens for regions under artificial selection in the sugar beet genome identified 15 Mbp of the genome as variation-poor, enriched for genes involved in shoot system development, stress response, and carbohydrate metabolism. The resources presented herein will be valuable for crop improvement and wild species monitoring and conservation efforts, and for studies on beet genealogy, population structure and population dynamics. Our study provides a wealth of data for in-depth analyses of further aspects of the beet genome towards a thorough understanding of the biology of this important complex of a crop species and its wild relatives.

## Introduction

Sugar beet (*Beta vulgaris* ssp*. vulgaris*) is one of the most intensively bred crop plants. It belongs to section *Beta* of Betoideae, a subfamily of the Amaranthaceae of the order Caryophyllales. Cultivation of beets in the Eastern Mediterranean may go back 10,000 years^1^. Initially, leaves were used for cooking. Today, the usage of beets covers a spectrum ranging from animal feed and bioethanol production to products for human consumption. Sucrose extracted from sugar beet covers around 14% of the world’s sugar needs^2^, and beet is the most important sugar provider after sugar cane. Sugar beet was domesticated from the wild sea beet *Beta vulgaris* ssp*. maritima*^1^, and new evidence indicates that the ancestors of sugar beet may have originated from Greek sea beets^3^. The development of beet lineages that accumulate high amounts of sucrose has occurred only recently during the last 200 years of intense breeding, which lead to an increase of sugar content from 8% in the sugar beet progenitor, the “White Silesian Beet”^4^, to 18% in extant sugar beets. Breeding has further led to high bolting resistance in sugar beets, as early flowering is a trait strongly disfavoured by beet farmers^1^. The bolting locus ("*B*-locus") is a well-characterized domestication target of beets^5^, encoding the *BvBTC1* gene which determines the beet life cycle. Beet crops uniformly harbour a *BvBTC1* allele which results in biennial plants. The biennial trait was selected from standing variation present in sea beets*,* where populations encompassing more biennial plants are found with increasing geographic latitude^6^. Its breeding history makes sugar beet a good model to study artificial selection acting on the genome in an early stage of domestication.

Three species are affiliated with the section *Beta* that can hybridise with each other: There are three wild taxa *B. macrocarpa*, *B. patula*, and *B. vulgaris*, whereby the latter is divided into the two wild subspecies *B. vulgaris* ssp. *maritima* and *B. vulgaris* ssp. *adanensis*, and the cultivated beet *B. vulgaris* ssp. *vulgaris* (comprising sugar beet, fodder beet, table beet, leaf beet/chard). The (sub)species within the genus *Beta* display different mating systems: The sea beet *B. v. maritima* and the crops derived thereof are, for most part, outcrossing. On the other hand, *B. macrocarpa* and *B. patula,* as well as *B. v. adanensis* are self-compatible^7^. The inbred nature of the genome of *B. patula* genome has facilitated assembling its genome using high-throughput sequencing data^8^. The molecular basis of self-incompatibility in beets is currently unknown^9^.

Species of the section *Beta* differ in their respective geographical distribution. It was hypothesised that the Messinian salinity crisis 5.96–5.33 million years ago, caused by the closing of the Strait of Gibraltar and subsequent drying up of the Mediterranean Basin, resulted in diversification of the genus *Beta*^10^. Today, sea beets (*B. v. maritima*) are found all along the Mediterranean coast, as well as along the Atlantic sea shores from Morocco up to Denmark. While sea beets predominantly inhabit coastal areas, reports of a Sicilian sea beet population thriving at an elevation of 1150 m demonstrate the remarkable adaptability of the species^1^. *B. v. adanensis* has its focus of distribution in the Eastern Mediterranean area, and mis-classification of *B. v. adanensis* as sea beet (*B. v. maritima*) has been observed^1, 3^. *B. patula* is a species endemic to the Madeira archipelago and *B. macrocarpa* is distributed widely in the Mediterranean area and Macaronesia^1^.

The possibility of genetic crosses makes sea beets and other wild *Beta* species interesting candidates to introduce biotic and abiotic stress resistances into beet crops to increase their yield.

Recently, hundreds of beets were analysed based on whole-genome sequencing data using a reference-free distance-based approach^3^. In this study, we extended the work by Wascher et al.^3^ and employed read mapping and variant calling relative to a reference genome, and used the identified genetic variants to investigate the dynamics of this comprehensive assemblage of beet accessions.

## Results

### *Beta* accessions analysed in this study

We analysed a large number of beet genomes on the molecular level making use of the more than 600 sequenced wild and cultivated *Beta* accessions published recently^3^ comprising *Beta vulgaris* ssp. *vulgaris* (mainly sugar beet, plus a few accessions described as fodder beet or leaf beet), *B. v. maritima* (sea beet), *B. v. adanensis*, *B. macrocarpa*, and *B. patula*. So far, genomic paired-end Illumina data of these accessions were analysed based on MinHash sketches resulting in global genomic distances that served as input for phylogenetic analyses^3^. While this reference-free approach was well suited for general measurements of genomic distances, any positional information was neglected as there was no mapping step to locate the sequencing reads in the genome. Here, we mapped the data against the sugar beet reference genome RefBeet-1.2^11^ and determined genomic variation along each chromosome. In addition to the accessions from the study mentioned above^3^ we included published data from the sea beet accession WB42^8^, another sea beet and five sugar beet accessions^11^, and one Swiss chard accession^12^ in our analysis. Furthermore, we analysed so far unpublished data of five *B. v. maritima* accessions (four obtained from the public repository of the USDA, one obtained from the Greek Genebank) and twelve *B. v. vulgaris* accessions (nine from the USDA, two from the East Lansing breeding program, one from IPK) (Table S1). We also included samples that had the same accession identifier (considered as duplicates) and had only been used for benchmarking so far^3^. In total, 656 sequencing data sets were processed (631 non-redundant accessions), and mapping results as well as variant calls for all of them are provided with this publication (see Data availability).

### Raw data and mapping

For the majority of accessions, the raw data comprised about 20 million read pairs per accession. Quality trimming reduced the average number per sample to 18.5 million read pairs corresponding to roughly sixfold genome coverage. Reads were mapped against RefBeet-1.2^11^ and filters for mapping quality and PCR duplicates were applied resulting in fivefold mapping coverage on average in the covered fraction of the reference genome. The lowest covered fraction was obtained for *B. macrocarpa* (average 38%), *B. v. adanensis* (58%), and *B. patula* (59%), as expected given the higher divergence of the wild beets relative to sugar beet that leads to more mismatches, and thus uncovered regions (Fig. S1). Sugar beet accessions covered on average about 75% of the reference sequence followed by Mediterranean sea beets (68%) and Atlantic sea beets (60%) reflecting the presumed origin of sugar beet from the area of Greece^3^.

### Genomic variation

**Figure 1:**
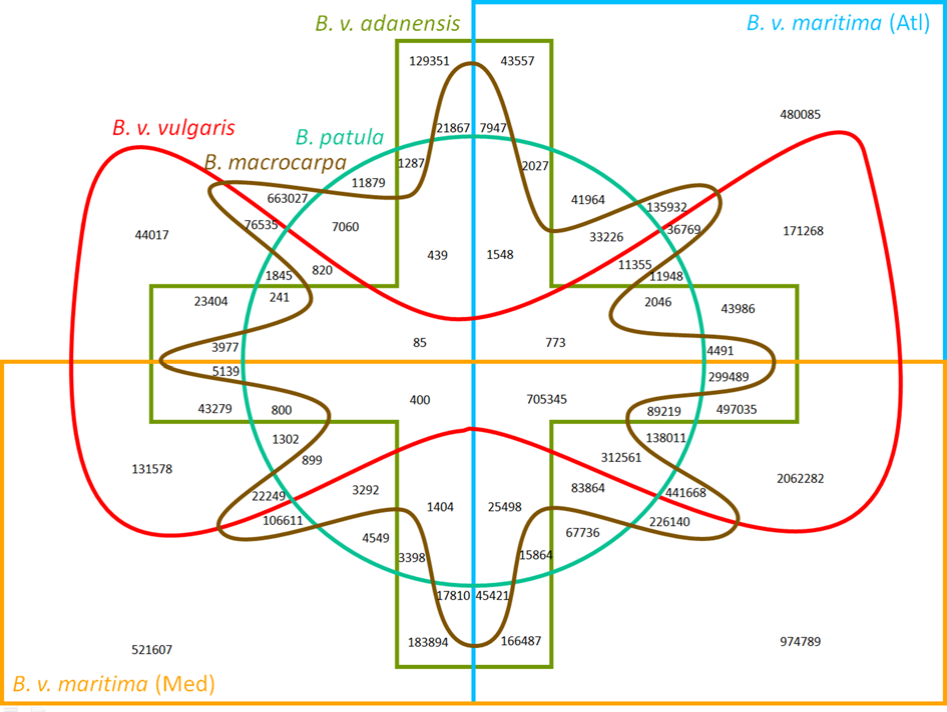
Venn diagram of shared and private single-nucleotide variants (SNVs) detected in accessions of the genus *Beta*. Sea beet (*B. v. maritima*) accessions are divided into two groups based on geographic provenance, i.e. *Atl* Atlantic region, *Med* Mediterranean area.

Single nucleotide variants (SNVs) and small insertions and deletions (indels) were determined relative to RefBeet-1.2 based on the mapped sequencing reads. Of about 70 million candidate variant positions we filtered for those that had at most 10% missing data (i.e. 90% of accessions covered a position by at least one read), were biallelic with a minor allele frequency larger than 1%, and were covered by 2 × N to10 × N reads where N is the number of covering samples at a position (for additional filters see “Methods”). The resulting 10,348,528 variant positions (9,255,888 SNVs and 1,092,640 indels with 622,333 deletions and 470,307 insertions) were kept for further analysis. With our filtering requirements we focused on genomic regions that were shared among most of the accessions so that variation in smaller subgroups may be underestimated. Summarised by species groups, *B. v. adanensis* showed more shared variation with Mediterranean sea beets than with Atlantic sea beets, whereas *B. patula* and *B. macrocarpa* shared more variation with Atlantic sea beets than with Mediterranean sea beets (Figs. 1, S2). The number of shared variants between *B. v. vulgaris* and sea beets was slightly higher in Atlantic sea beets than in Mediterranean sea beets if only SNVs were considered and higher in Mediterranean sea beets when short indels were also considered. When analysing the number of variant positions by chromosome we observed similar rates for each reference chromosome except for chromosome 6. Chromosome 6 showed generally higher and more lineage-specific variation supporting findings of a previous study^13^.

### Variant-based clustering

**Figure 2:**
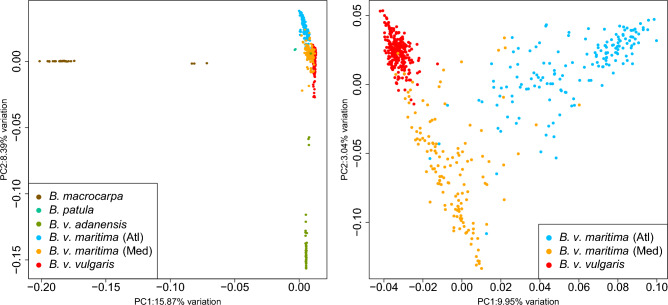
Principal component analysis (PCA) for accessions of the genus *Beta* (left), and for sea beets and sugar beets only (right). Sea beet (*B. v. maritima*) accessions are divided into two groups based on geographic provenance, i.e. *Atl* Atlantic region, *Med* Mediterranean area.

The genotypes called at variant positions were used as input for a principal component analysis (PCA) to detect clusters of samples based on shared variation. Additionally, we performed clustering based on genotype likelihoods inferred from read mapping results using ANGSD^14^. Although the number of variant positions differed substantially due to the filtering settings in each approach (10.3 million variants vs. 3.7 million variants, respectively) the outcome showed similar clustering results (Figs. 2, S3). Sea beets and sugar beets clustered together whereby Atlantic and Mediterranean sea beets were separated in subclusters and sugar beets had their own dense subcluster in close vicinity to Mediterranean sea beets. This clustering was even more obvious when analysing sea beets and sugar beets separately from the other wild beets species (Figs. 2, S4). *B. patula* accessions clustered together close to the sea beet cluster. We could clearly identify a cluster of *B. macrocarpa* and a cluster of *B. v. adanensis* accessions, both distinct from each other and from the remainder of samples. Both species showed a few accessions that were separated from their main cluster (for *B. macrocarpa*: BETA 572, BETA 709, BETA 881; for *B. v. adanensis*: BETA 591, BETA 1405, PI 604518, PI 604545), perhaps representing intermixed or hybrid individuals. A fourth accession, BETA 18, classified as *B. v. maritima* but described as outlier previously^3^ was found close to the three *B. macrocarpa* outliers in a chromosome-wise PCA, namely in chromosomes 2, 6, and 7 (Fig. S5). Also, some *B. v. maritima* accessions clustered closer to *B. v. adanensis* in chromosome-wise PCA plots. Analysing only sugar beets in a separate PCA showed varying clouds for each chromosome but showed no clear patterns that would correlate with known groups of accessions, e.g. different breeding sources.

### Admixture analysis and phylogenetic trees

For a reduction of variant positions to be analysed we tried two different approaches, one focusing on exonic 4DTv sites and one on intergenic variants. Either of these variant subsets primarily consists of substitutions that are selectively neutral, and phylogenetics analyses based on such data should lead to similar results.

For variant-based analyses one has to find a set of genomic positions that is suitable to be analysed in all 656 samples comprising different species and subspecies. Due to heavy filtering towards the selection of shared genomic regions there may be biases that impact genome-wide conclusions. For variant-based tree calculation we chose a maximum likelihood approach as implemented in IQ-TREE^15^ based on 50,124 4DTv exonic sites and 71,771 intergenic sites, respectively. The resulting trees were inspected regarding their overall topology, the formation of subtrees corresponding to (sub-)species assignments and geographic origin, and the placement of previous and newly emerging outliers.

The main separations were confirmed in both trees, i.e. separating sea beets from sugar beets, showing *B. macrocarpa* and *B. patula* as most distant species close to the root of the tree, and clustering *B. v. adanensis* accessions together (Figs. S6, S7). However, the placement of the *B. v. adanensis* subtree was either within the group of Mediterranean sea beets (tree based on exonic variants) or at the root of the subtree of Mediterranean sea beets (tree based on intergenic variants). Accessions that were outliers in one tree (e.g. BETA 156 or PI 518,320 in the tree of intergenic variants; PI 546,414 in the tree of exonic variants) were integrated in their expected subtree in the other tree. BETA 18, previously found as outlier^3^ and showing a separate placement in three chromosomes in the PCA (see above), was placed either at the root of the *B. macrocarpa* subtree (exonic) or at the root of the Atlantic sea beet subtree (intergenic), with bootstrap support of 100% in each case. Among the sugar beets obtained from breeders only Strube lines were clustered together consistently. We concluded that an analysis other than tree calculation may be more informative and performed an admixture analysis.

**Figure 3:**
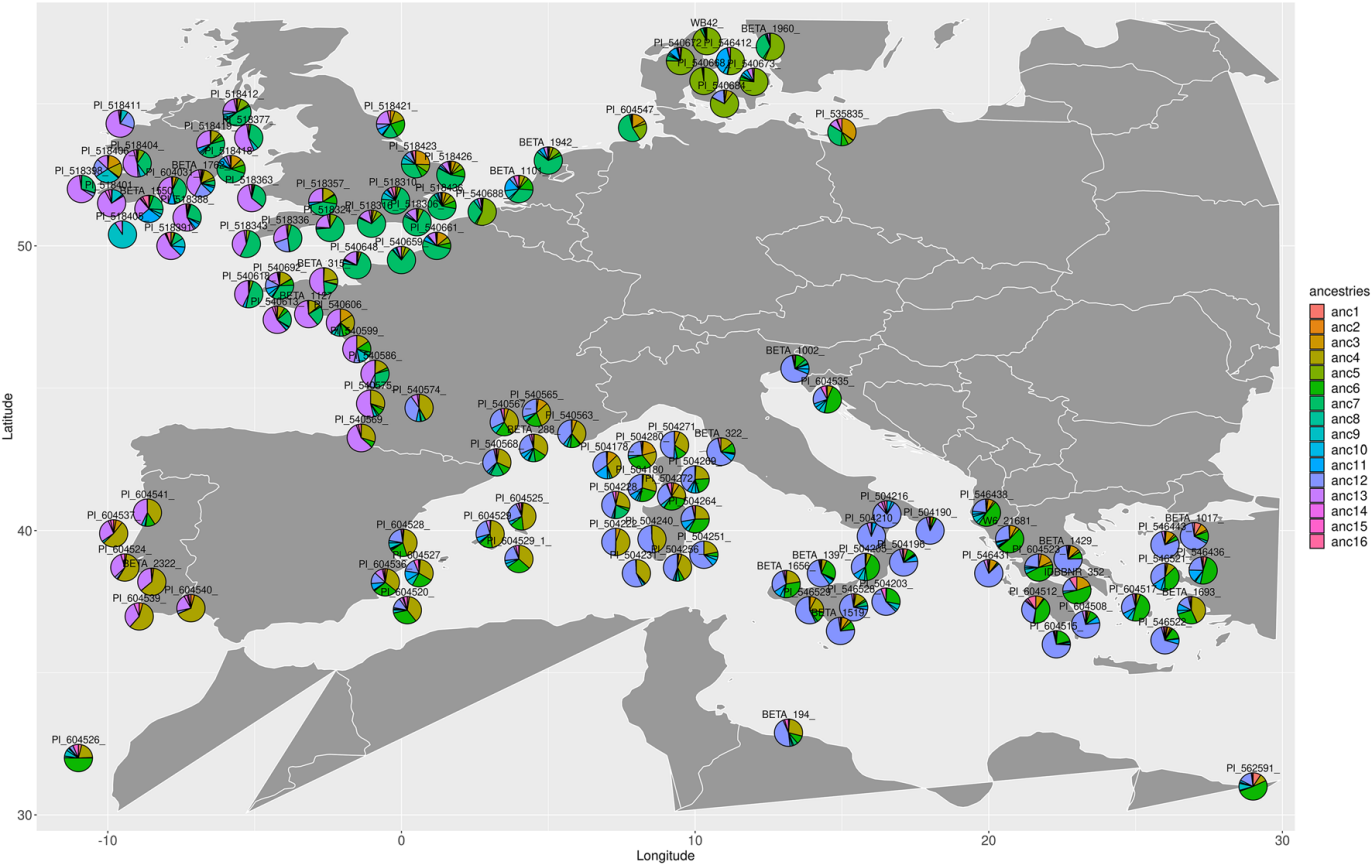
Admixture pie charts of selected sea beet accessions based on exonic variants (k = 16). Geographic coordinates were adapted for better visibility. The full panel of wild beet accessions with geographic information available is shown in Fig. S8.

Filtering of variant positions for admixture analysis started from the same ~50 thousand exonic variants that were selected for tree calculation or from 3.8 million intergenic variants, respectively, resulting in 36,634 exonic variants and 234,716 intergenic variants, respectively. According to the Evanno method^16^ a *k* of 16 best captured the variation observed in the exonic data and a *k* of 12 best captured the variation observed in the intergenic data. We displayed admixture bar plots for both data sets along the phylogenetic trees (Figs. S6, S7) and pie charts for the exonic data on a geographic map (Figs. 3, S8). The admixture plots revealed separated ancestries for *B. macrocarpa* ("anc2" in Fig. S8), *B. patula* ("anc14"), and *B. v. adanensis* accessions ("anc1"), and extensively intermixed ancestries for *B. v. maritima* and *B. v. vulgaris* accessions. Accessions at the root of the *B. macrocarpa* subtree showed intermixed ancestries of *B. v. maritima* and *B. macrocarpa*, and, likewise, accessions at the root of the *B. v. adanensis* subtree had intermixed ancestries of *B. v. maritima* and *B. v. adanensis*. We assume that these accessions represent hybrids that were formed after speciation of the different wild beet (sub-)species. The placement of the *B. v. adanensis* subtree relative to *B. v. maritima* subtrees (see above) may be explained by such hybrid accessions that appear as transition between the two subspecies during tree calculation although the distances between genuine *B. v. maritima* and *B. v. adanensis* support an earlier separation and independent development. Ancestry bar plots were in line with the tree topologies in most cases, and several subtrees showed their own dominating ancestry or ancestry proportions. The outliers mentioned above showed admixture plots that differed from their neighbouring accessions in the tree reflecting the difficulty to place them correctly in a tree format. BETA 18 showed a considerable fraction of *B. macrocarpa* ancestry ("anc2" in Fig. S8; plotted in the area of former Yugoslavia according to passport data), explaining its placement as *B. v. maritima* outlier close to *B. macrocarpa* in PCA plots and trees. We found that resolving the admixture information on a geographic map showed the relation of accessions more clearly than the trees. Intermixed ancestries of *B. v. adanensis* and *B. v. maritima* appeared in the Eastern part of the Mediterranean area (where *B. v. adanensis* is typically found). There was a characteristic ancestry for Mediterranean sea beets (blue-violet, "anc12" in Fig. 3) and another ancestry (purple, "anc13") along Portugal and Northern France up to the British Isles that was characteristic to Atlantic sea beets. Another ancestry (light green, "anc7") dominated further northwards, from the coasts of the Normandy and Eastern Britain towards northern Germany, and finally, in the most northern area of sea beet distribution, the Danish sea beets had their own characteristic ancestry (light olive, "anc5").

### Signatures of artificial selection

We expected to find genomic regions of low diversity in sugar beets (“variation deserts”) due to artificial selection. Such regions most likely represent signatures of breeding and are expected to contain domestication genes. We restricted the analysis to accessions with low variability at the bolting locus (*B*-locus), a well-studied domestication gene of beets^5, 17^ encoding the *BvBTC1* gene which determines an annual vs. a biennial life cycle. We observed two clusters of variation at the *B*-locus (10,338 bp, *BvBTC1* gene including introns), most accessions had zero to five variants at the *B*-locus and for the rest of the accessions 16–47 variants were observed. Two hundred and six sugar beet accessions that clustered within the monophyletic sugar beet clade (according to the tree shown in Fig. S7) and had a maximum of five variants at the *B*-locus were used for variation desert analysis. Additionally, we analysed a subset of 54 Mediterranean sea beets to exclude deserts that were not unique to sugar beets and thus could not be attributed to domestication nor breeding. Variation along RefBeet-1.2 was analysed in windows of 2 kbp length shifted by 1 kbp, and windows that were well covered but showed no more than two variants per sugar beet accession were considered as variation desert (see “Methods”).

**Figure 4:**
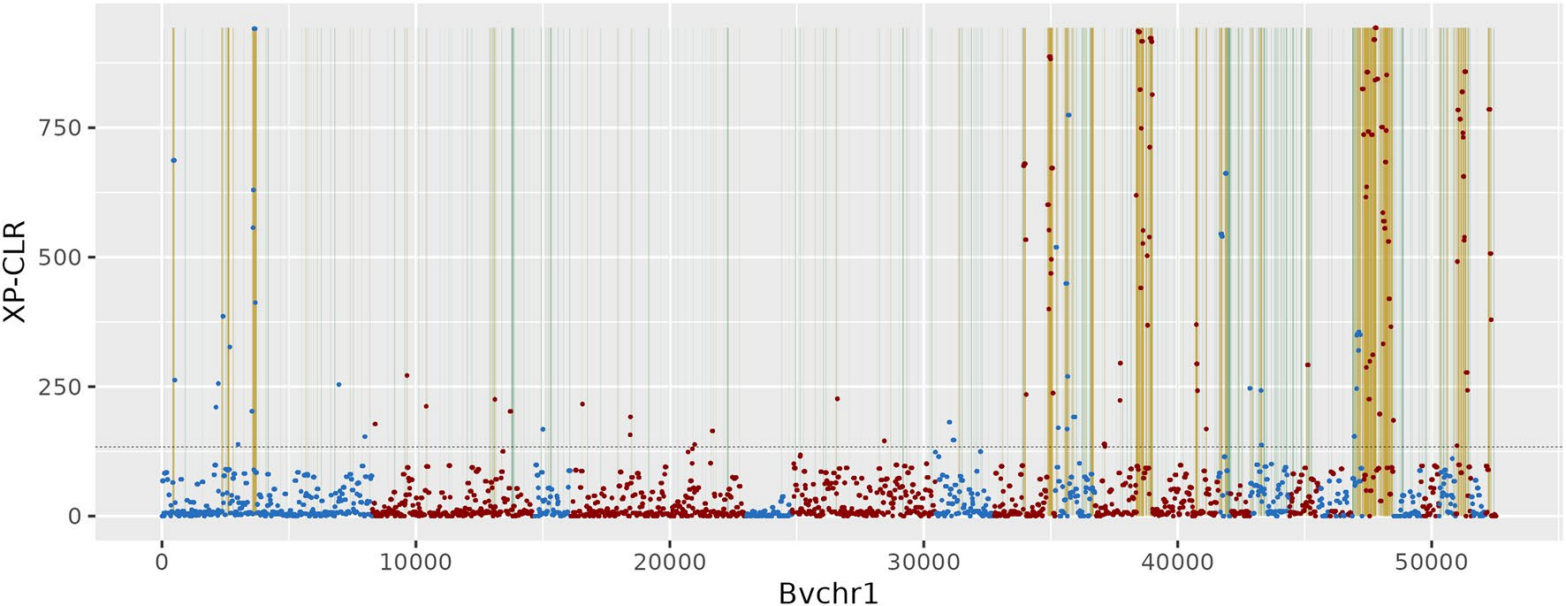
Variation-poor regions in sugar beet chromosome 1 using a sliding window approach and a cross-population composite likelihood ratio test (XP-CLR). Green vertical bars indicate low variation in 1 kbp windows, red and blue data points (alternating by scaffold) represent XP-CLR values along RefBeet-1.2 (tics in kbp), regions of high XP-CLR values are highlighted in yellow. The horizontal line marks the 95%ile of XP-CLR values.

In total, we identified 40.2 Mbp and 38.6 Mbp desert windows in sugar beets and sea beets, respectively, of which 15 Mbp were shared such that 25.2 Mbp private sugar beets deserts remained for further analyses. Deserts that were separated by up to1 kbp were merged, resulting in a total of 26 Mbp of private sugar beet variation deserts corresponding to 6219 separate regions. These regions were in general evenly distributed between and along chromosomes with an average length of 4.2 kbp and a maximum length of 86 kbp (Figs. 4, S9 and Table S2). Of a total of 2375 genes with a mean length of 7.8 kbp that were at least partially located inside variation desert windows, 736 genes (31%) were inside a desert window with at least 90% of their length and 1375 genes (58%) were completely inside a desert as the only gene in this desert.

As a complementary method we performed a cross-population composite likelihood ratio test (XP-CLR)^18^ based on allele frequencies. XP-CLR is most sensitive to recent selection and can detect deviation from neutrality at sites close to a beneficial allele. The analysis was performed for sliding windows of size 50 kbp with 10 kbp step-size and the top 5% of windows with the highest normalised population differentiation statistic XP-CLR score were extracted, which summed to a total size of 66 Mbp. We observed an overlap of 15 Mbp between the top 5% XP-CLR windows and private sugar beet variation deserts.

**Figure 5:**
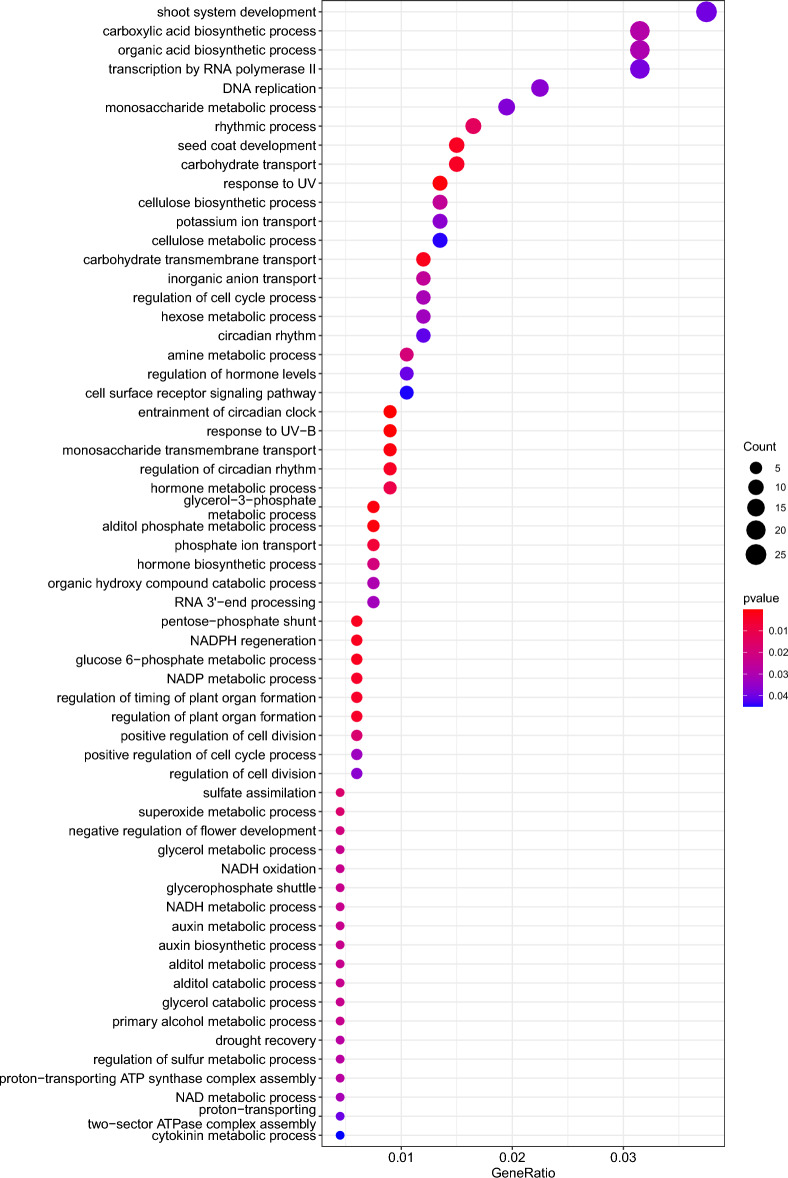
Biological processes of enriched genes located in variation deserts private to sugar beets and identified as differentially selected with XP-CLR.

Finally, 1317 genes that were at least partially contained within variation deserts that overlapped with the top 5% of XP-CLR windows were used for gene ontology (GO) term enrichment analysis (Fig. 5, Table S3). We found significantly enriched GO terms involved in biological processes that are of interest to breeders, like response to abiotic stress factors, detoxification, flower development, shoot system development, and sugar transport (Fig. 5, Table S3). In order to validate these findings, we selected two of the genes identified during this analysis. First, we chose Bv2_025460_xhyw encoding the ultraviolet-B receptor gene UVR8. The UVR8 gene has not been studied in beets yet, but evidence from other species including *Arabidopsis* identifies this gene as a growth regulator in plants under stress induced by UV-B radiation^19^. Intriguingly, sugar beets were fixed for the reference haplotype with few exceptions, while much variation was observed among the different wild beet accessions used in this study (Fig. S10), providing confirmation that artificial selection has been acting upon the UVR8 locus in sugar beet. As another example we chose Bv3_054860_wjth, encoding the inositol transporter 1 gene. The role of inositol in freezing tolerance in sugar beet has been described previously. Briefly, inositol is required for the synthesis of the trisaccharide raffinose, that acts as a cryoprotectant in taproot tissue^20^. Again, sugar beets were mainly homozygous for the reference haplotype, while more variation was observed among wild beet accessions (Fig. S11).

## Discussion

We determined the genetic variation in species of the genus *Beta* on the sequence level based on a large number of completely sequenced genomes. The data were compared to the sugar beet reference genome RefBeet-1.2, and about 10 million variant positions were detected and analysed. Most previous studies either relied on a small sample size or on a small number of genetic markers that were investigated. A recent study employed a large number of genomes but only performed reference-free analyses^3^. While there were many interesting findings in the reference-free approach we here show how including a reference can go beyond k-mer-based analyses. The diversity of the species can be quantified in terms of shared variation relative to a single reference genome as a comparable measurement among all accessions in contrast to the pairwise results of the reference-free approach. Furthermore, the analyses can be separated by chromosome to detect differences in the distribution of the variation. We identified regions of high and low variation along the reference genome, located genes in such regions, and analysed their functions. Further subsetting may be performed to narrow down the regions and genes that distinguish subgroups from each other.

For variant-based tree calculation there may be the disadvantage that only positions that are covered by all accessions can be included. In this way, regions of genetic importance to some groups of accessions may be missed, and the tree topology may be less reliable. For example, we see a placement of the *B. v. adanensis* subtree in the variant-based trees that is different from the placement in the k-mer-based tree. However, the variant-based trees that placed *B. v. adanensis* either between Mediterranean and Atlantic sea beets or within the Mediterranean cluster of sea beets included all samples, whereas the k-mer-based tree focusing on wild beets contained only a small number of accessions. According to the distances of the *B. v. adanensis* branches we still believe that *B. v. adanensis* separated early from the common ancestor and only later came in contact with *B. v. maritima* again leading to hybrids that are also contained in our data set and confuse calculations in a strictly bifurcating tree structure. In fact, the initial tree of the k-mer-based approach showed such hybrid accessions (as determined later on) as subtree within the Mediterranean cluster of sea beets as well^3^. Further work on the history of beet evolution needs to be carried out to uncover their relationship in more detail.

Using the variant positions we could perform an admixture analysis that shed light onto the assumed ancestry proportion in every single accession and to reveal their relationships more clearly than in the trees. Indeed, there was a sharply distinguished ancestry found in *B. v. adanensis* accessions indicated by a proportion of almost 100%. This ancestry was found at proportions of around 25–75% in accessions that were considered *B. v. maritima* but clustered close to *B. v. adanensis* in the trees. From the ancestry proportion of *B. v. maritima* accessions, especially when generating admixture pie charts, it was most easily to grasp which ones contained a considerable amount of the *B. v. adanensis* ancestry and which ones seemed to be not admixed with *B. v. adanensis*.

Admixture analysis further revealed how ancestry proportions changed over the entire range of *B. v. maritima* distribution that was covered in this study. Most prominently distinguishable was the group of Danish accessions indicating their specific adaptation at the edges of the species range. The admixture proportions further confirmed the separation of Mediterranean and Atlantic sea beets with specific ancestries and only one shared ancestry found in both subgroups.

We conclude that in our setup the reference-free tree calculations were well suited for an overview of the phylogeny, whereas reference-based variation was most valuable for locating specific differences in the genome, to identify genes involved in phenotypic differences, and to perform insightful admixture analyses.

Genomic regions in the reference that showed little variation throughout a large number of sugar beet accessions indicate which parts of the genome were conserved, most likely as a result of breeding efforts. The genes contained in these regions may have played a role during beet domestication and breeding as suggested by the enriched GO terms pointing to important traits. As reported in a previous study comparing a single sea beet to the reference genome^11^, we still found a considerable fraction of shared regions that showed little variation when comparing 54 sea beets to the reference. Considering that the species is outcrossing with a generally high level of heterozygosity it is unexpected to find a substantial proportion of shared low-variation regions in the genomes. The genes contained in such regions may be interesting targets for further studies.

Our data establish a broad basis to further explore the genetic architecture of the beet genome, for both the cultivated and the wild (sub)species, and provide future perspectives for introduction of useful traits into commercial breeding lines.

## Conclusions

We provide data of hundreds of beet genomes mapped to a reference genome and covering the genetic variation in several species of the genus *Beta*. Our analyses reveal variant-based clustering of cultivated and wild beets into (sub)species using different approaches and locate genomic regions of low variation within the genome of sugar beet. The genes in such regions are enriched for functions related to important traits. We provide examplary evidence that two of these genes indeed were most likely targets of artificial selection by showing that sugar beets almost exclusively are homozygous for one haplotype. To date, our data represent the most comprehensive genomic resource of the genus *Beta* for research and breeding activities.

## Methods

### Plant material and sequencing

In addition to 606 accessions of the genus *Beta* previously analysed by Wascher et al. by comparing pairwise genomic distances, the current study used further 17 beet accessions obtained from the USDA (13 accessions), East Lansing sugar beet breeding program (2 accessions), IPK Genebank (1 accession), and from the Greek Gene Bank (1 accession). DNA extraction and sequencing were performed as described^3^. We also included eight accessions of beets with assembled genomes, including five sugar beets^11^, the sea beets DeKBm^11^ and WB42^8, 21^, as well as the chard genotype M4021^12^. Accessions are listed in Table S1, along with their provenance (public seed bank, plant breeding company). All wild beet accessions were from public sources, and the plant material had been collected prior to 2014 when the Nagoya protocol came into effect.

### Raw data processing and mapping

Quality filtering and trimming was done using *Trimmomatic* v.0.35^22^ with the following settings: ILLUMINACLIP:illumina.adapter.fa:2:30:10 LEADING:28 TRAILING:28 SLIDINGWINDOW:5:15 MINLEN:50. *FastQC*^23^ was used to generate quality reports. The sequencing reads were mapped to the sugar beet reference genome RefBeet-1.2^11^ with *bowtie2* v.2.3.4.1^24^ using mapping parameters that were optimised for the observed insert sizes (-D 20 -R 3 -N 1 -L 20 -i S,1,0.5 -gbar 1 -mp 4 -I 250 -X 1500). *samtools* v.1.12^25^ was used to sort the data (*samtools sort*) and remove PCR duplicates (*samtools rmdup*) and to filter for mapped read pairs with a minimum mapping quality of 20. *picard* v.2.3.0^26^ was used to add read groups to the mapping files (AddOrReplaceReadGroups, example: RGID = 1 RGLB = SAMPLE1 RGPL = illumina RGPU = SAMPLE1 RGSM = SAMPLE1), which were then indexed with *samtools*. *bedtools* v.2.25.0^27^ was used to calculate the genome coverage per site, which was then averaged per chromosome and the total size of the RefBeet-1.2 assembly. Coverage per site was calculated using *bedtools genomecov* -d.

### Genomic variation

*gatk HaplotypeCaller* (setting -emit-ref-confidence GVCF), *CombineGVCFs*, and *GenotypeGVCFs* v.4.1.0.0^28^ were used for variant calling over all samples. *gatk SelectVariants* was further used to filter the resulting multi variant calling file for low quality calls using a modification of *gatk* best practices parameter settings (-select "MLEAF > 0.01" -restrict-alleles-to BIALLELIC -max-nocall-fraction 0.1 -select "MQ > 30" -select "DP > 1344" -select "DP < 6720" -select "SOR < 3" -select "QD > 2" -select "FS < 60" -select "MQRankSum > − 12.5" -select "ReadPosRankSum > − 8" -select "BaseQRankSum > − 12.5"). Numbers of shared variants were determined at^29^ with the setting "Non-Symmetric" shape.

### Variant-based clustering

PCA was performed with *smartPCA*^30^. The genotypefile, SNVfile, indfile and parfile needed to run *smartPCA* were generated with *bcftools query*^31^ and custom *bash* and *python* scripts. Results were visualised using *R*^32^.

We generated an identity-by-state (IBS) matrix with *ANGSD* v.0.930^33^ using the whole-genome mapping results and the following parameters: -uniqueOnly 1 -remove_bads 1 -doMajorMinor 1 -GL 2 -doMaf 1 -minMaf 0.01 -minMapQ 30 -minQ 20 -SNP_pval 1e−6 -minInd 634 -doIBS 1 -doCounts 1 -setMinDepth 1294 -setMinDepthInd 2 -makeMatrix 1 -doCov 1. The pairwise distances (Fig. S3) were visualised using the *R* package *heatmap*.

### Admixture analysis and phylogenetic trees

We calculated maximum likelihood (ML) trees with *IQ-TREE v.2.1.3*^15^ based on genome-wide four-fold degenerated (4DTv) sites and based on intergenic variants. *Annovar*^34^ was used to extract 4DTv variants and intergenic variants based on the RefBeet-1.2 annotation. First, the *bioconda* package *gff3ToGenePred*^35^ was used to convert the RefBeet-1.2 annotation from .gff to .genepred format. The *Annovar* *Perl* scripts *Retrieve_seq_from_fasta.pl* (-format refGene) and *annotate_variation.pl* (-geneanno -dbtype refGene) were then used to extract the wanted variants from the variant calling file, which were concatenated to a multi-fasta format file with *bcftools* v.1.3.1^31^, *python* and *bash*. The intergenic variant dataset was reduced and linkage disequilibrium (LD) considered by requiring a minimum distance of 1 kb between variants. Heterozygous variants were provided as ambiguity codes. The best substitution model (TVM + F + ASC + R10 for the 4DTv dataset and SYM + R10 for the intergenic dataset, respectively) was automatically chosen by the program according to the Bayesian Information Criterium (BIC). The consensus of a total of 1000 bootstrap replicates was annotated onto the best-scoring ML tree (Figs. S6, S7), which was midpoint rooted and visualised with *figtree* v.1.4.2^36^.

We used *PCAngsd* v.0.981^37^ to calculate ancestry profiles assuming *k* ancestral populations ranging from *k* two to 60 for the whole dataset. The site allele frequency spectrum was first calculated using the whole-genome mappings as input for *ANGSD*. We performed multi-sample genotype likelihood estimation (-doSaf 1) according to *GATK* (-GL 2) and provided the RefBeet-1.2.fa reference as reference and ancestral fasta (-anc file.fa -ref file.fa) to obtain a beagle likelihood file (-doGlf 2). Major and minor allele were inferred from the genotype likelihoods (-doMajorMinor 1). Per-site frequencies were calculated with fixed major and minor allele (-doMaf 1). Only sites with minor allele frequency (MAF) higher than 1% (-minMaf 0.01) and with a SNP p-value smaller than 1e−6 were considered (-SNP_pval 1e−6). We counted base frequencies (-doCounts 1) that had a minimum base quality of 20 (-minQ 20) and required a minimum of 90% of the individuals to cover a site (-minInd 0.9*N, where N is the number of samples) with at least two reads (-setMinDepthInd 2) to be considered in the analysis. Of all variants that made it through these filters, we kept only those that were 4DTv or intergenic sites, respectively (-sites file.bed). The beagle output files were used as input for *PCAngsd* to calculate the admixture proportions (-admix). The seed for initialization of factor matrices (-admix_seed) was set to values ranging from ten to 50 with step size ten to test for convergence of five independent runs per tested *k* (-admix_K). The *k* best explaining the variance observed in the data was evaluated using the Evanno method^16^ as implemented in *CLUMPAK*^38^ by comparing log probabilities per run per *k*. *Python* was used to convert resulting admixture files (*.Q.npy) from numpy to a format readable by *R* for visualisation. Results were aligned to the phylogenetic tree using the *R* package *ggtree*^39^ and finalised with *GIMP*^40^. Pie charts (Figs. 3, S8) were generated using *ggplot*^39^ and *geom_scatterpie* from the *R* package *scatterpie* v. 0.1.7.

### Signatures of artificial selection

Variant calling files for each of the two tested populations (sugar beet vs. Mediterranean sea beets) were extracted with *gatk SelectVariants* from the previously generated file including all samples. To account for reference and sequencing errors, variants with MAF < 0.05 were excluded. Per population, a custom *python* script was used to print the number of SNVs and indels detected in 2 kbp sliding windows (1 kbp step-size) of RefBeet-1.2. *bedtools*, *bash* and *python* were used to get the mapping depth, mapping width, and the percentage of samples with missing data per window and population. Finally, filters were applied to each window according to observations in the *B*-locus: max. two variants (max. four in sea beets, based on the genome-wide median of variants per 2 kbp window), min. mapping depth six (min. four in sea beets), min. 90% of window covered by reads (80% for sea beets) and min. 90% of the data have reads mapped to the window. Slightly different filters for sea beet than for sugar beet were chosen based on their lower coverage.

To confirm identified variation deserts an allele-frequency-based method called cross-population composite likelihood ratio test (XP-CLR)^18^ was undertaken. The analysis was performed for sliding windows of size 50 kbp with 10 kbp step-size, and the top 5% of windows with the highest normalised population differentiation statistic XP-CLR score were extracted.

Genotypes of two selected genes (Figs. S10, S11) were plotted using the *R* function *pheatmap* v1.0.12.

### Gene ontology (GO) term enrichment

We performed GO term enrichment analyses of genes that were located within variation deserts and identified as differentially selected regions with XP-CLR. First, we generated an organism database (OrgDb) for *Beta vulgaris* using the *R* package *AnnotationForge*^41^ that subsequently served as gene universe for the GO term enrichment analysis with *clusterProfiler*^42, 43^. Before generating the OrgDb it was necessary to extract for each gene the codon sequence from the RefBeet-1.2 .gff file and convert it to amino acid code using *gffread*^44^. The resulting fasta files were used to perform *BLASTP* v.2.9.0 searches^45^ against the *NCBI* nr database (-taxids 3555 -outfmt 14). *BLASTP* results were used as input for *OmicsBox* v.2.0 (https://www.biobam.com/omicsbox/) to perform GO mapping and *blast2go* annotation^46^ to finally get the input for OrgDb generation. To generate the query, gene names and respective GO terms of annotated regions that overlapped with or were part of variation deserts were first extracted from the RefBeet-1.2 .gff file using *samtools*. A *BLASTN* search^ 45^ of candidate genes against the *NCBI* nt database was conducted (-perc_identity 90) and RefSeq IDs of the best hits were extracted, which were needed as query against the gene universe for GO term enrichment analysis. After loading the gene universe and the query into *R*, the query RefSeq IDs were converted to GID and GOALL (extracted from gene universe) using the function *bitr* from the package *clusterProfiler*. Finally, *enrichGO* from the package *clusterProfiler* was used to perform GO term enrichment analysis of genes hit by private sugar beet variation deserts for each gene ontology category separately (pvalueCutoff = 0.05, qvalueCutoff = 1 and pAdjustMethod = "none"). *dotplot* and *plotGOgraph* were used for visualisation of the results and GO terms with p < 0.05 were extracted.

### Computing resources

Data were processed on a high-performance Linux computing cluster featuring a CentOS 6.7 and CentOS 7 operating system with nine computing nodes each equipped with up to 56 cores (112 logical CPUs) and up to 1 TB of RAM.

### Scripting, data visualisation and plotting

*R* analyses were run with *R* v.4.0.5^32^. Perl scripts were run with *Perl* v.5.10.1. Custom scripts were coded with *Bash* v.5.0 and *Python* v.3.6.

## Supplementary Information

Supplementary Information 1.

Supplementary Table S1.

Supplementary Table S2.

Supplementary Table S3.

## Data Availability

Raw data generated in this study are available at the NCBI SRA under accession numbers SAMN30181342-SAMN30181359 (BioProject PRJNA815240). Mapping files and variant calls are provided at http://bioinformatics.boku.ac.at/Download/BetaVariation/.
