# Supplementary material for: Genomic variation in the genus Beta based on 656 sequenced beet genomes
Source: Sci Rep. 2023 May 27;13:8654. doi: 10.1038/s41598-023-35691-7 (PMC10224960; doi:10.1038/s41598-023-35691-7)
Supplement: Supplementary file 1 — Supplementary Information 1. [file 41598_2023_35691_MOESM1_ESM.pdf]

Supplementary Information

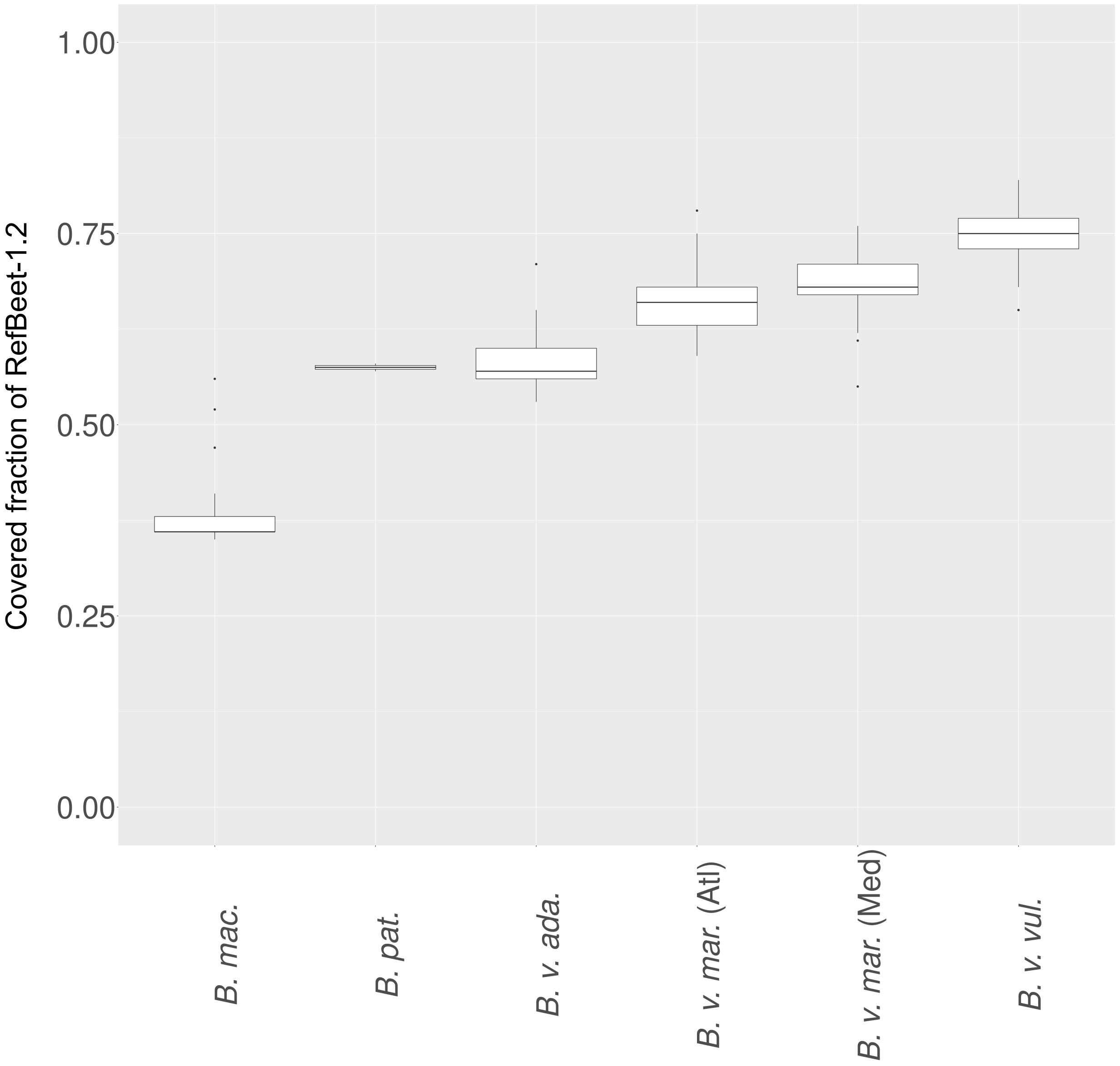

Figure S1: Covered fraction of the sugar beet genome assembly RefBeet-1.2 with sequencing reads from *Beta* accessions grouped by species or subspecies. Percentage of RefBeet-1.2 covered by at least one read after filtering is indicated. *B. mac.*: *Beta macrocarpa*; *B. pat* : *Beta patula*; *B. v. ada.*: *Beta vulgari*s ssp. *adanensis*; *B. v. mar*. (Atl): *B. vulgaris* ssp. *maritima* (Atlantic region); *B. v. mar*. (Med): *B. vulgaris* ssp. *maritima* (Mediterranean area); *B. v. vul.*: *B. vulgari*s ssp. *vulgaris*.

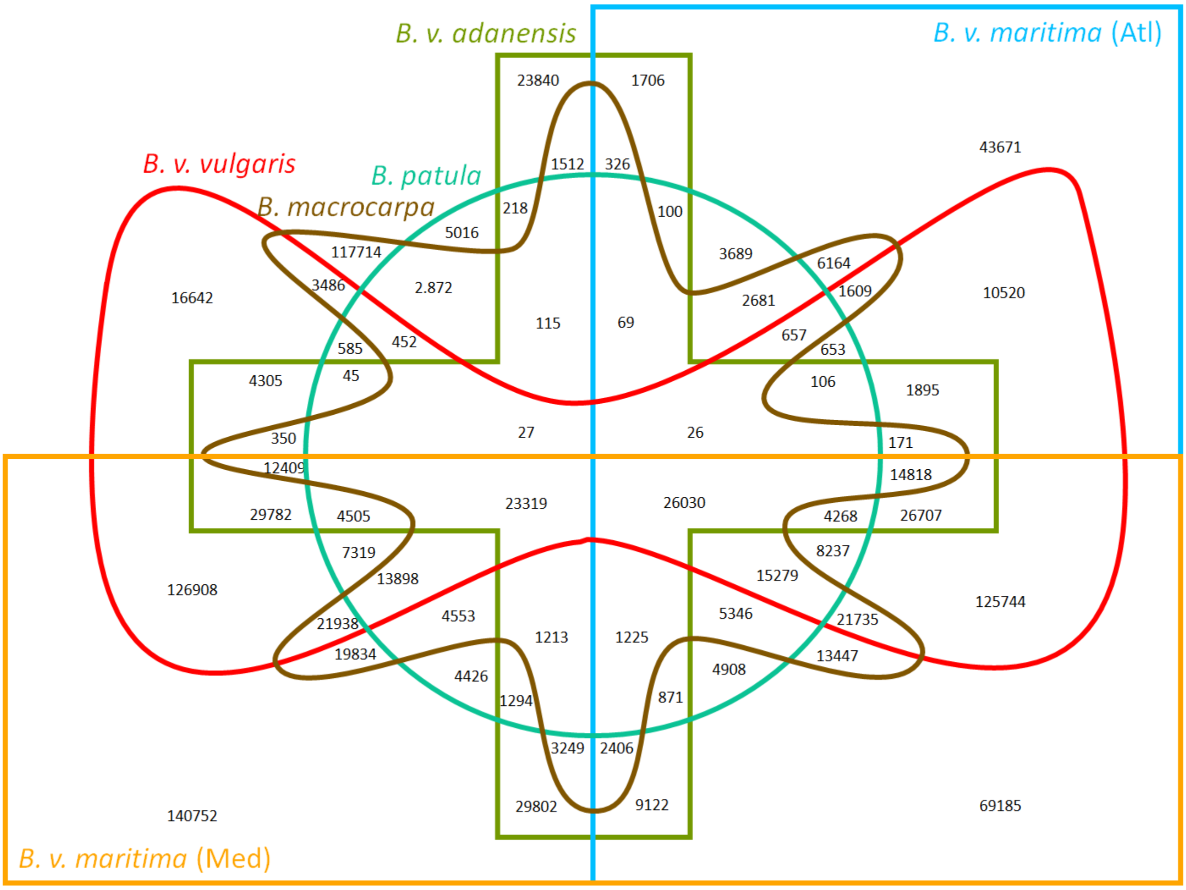

Figure S2: Venn diagram of shared and private insertion/deletion variants (indels) detected in the genus *Beta*. Sea beet (*B. v. maritima*) accessions are divided into two groups based on geographic provenance, i.e. Atl: Atlantic region; Med: Mediterranean area.

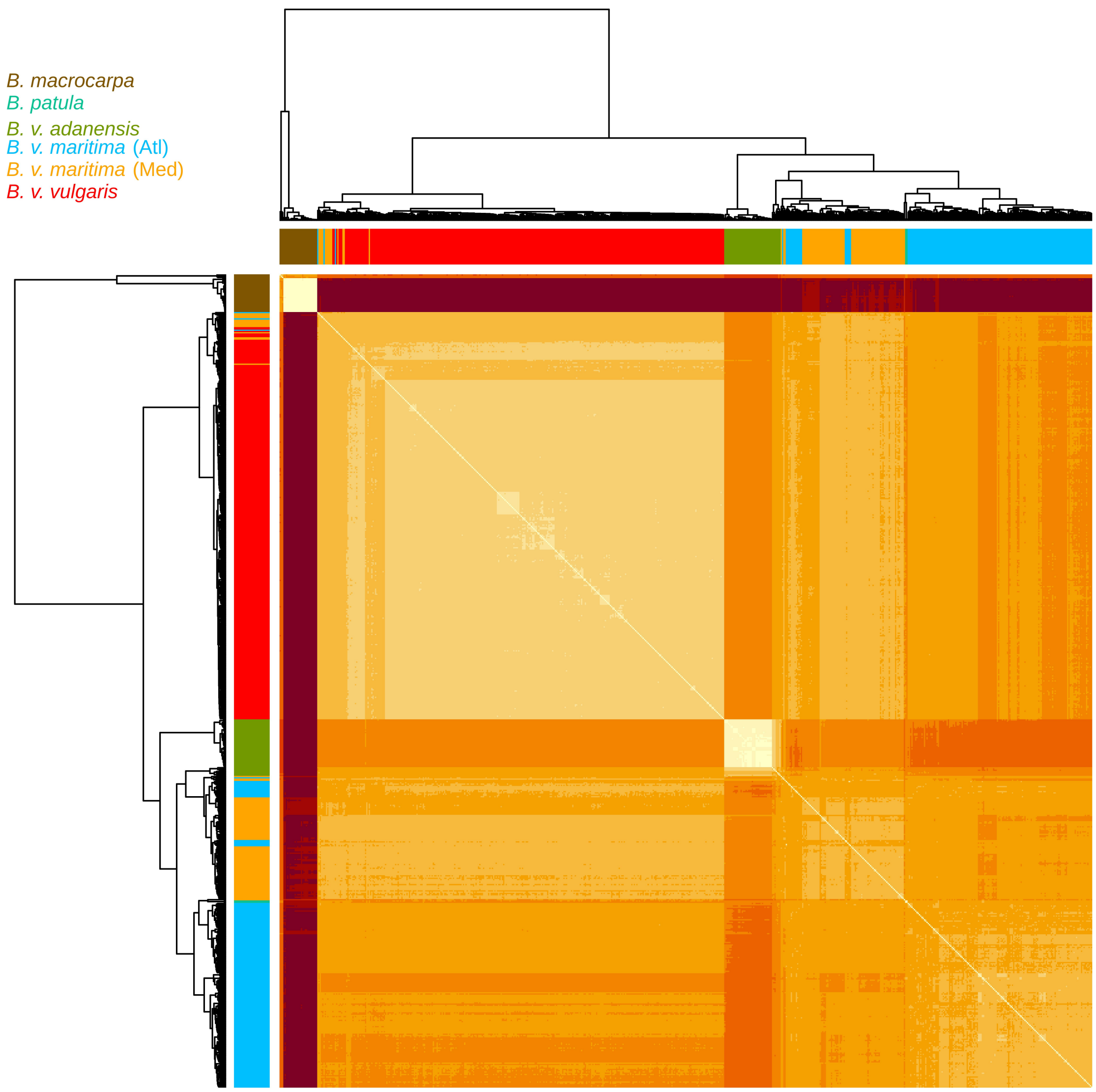

Figure S3: Heatmap and dendrogram based on pairwise identity-by state (IBS) distances obtained from variants identified in beets relative to the RefBeet-1.2 sugar beet reference genome sequence. Sea beet (*B. v. maritima*) accessions are divided into two groups based on geographic provenance, i.e. Atl: Atlantic region; Med: Mediterranean area. Distance increases from white to red.

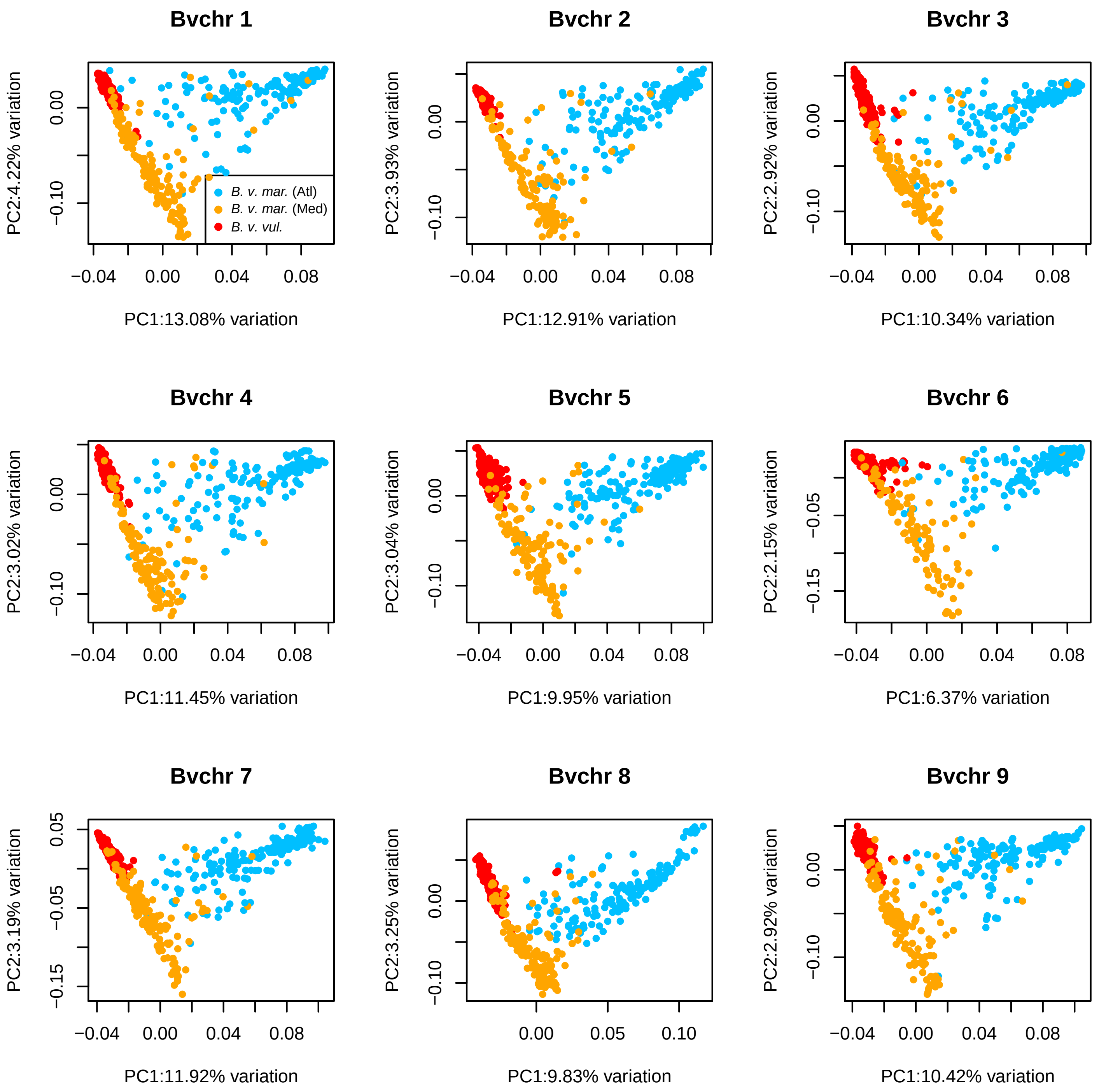

Figure S4: Principal component analysis (PCA) for the nine beet chromosomes, based on variants of sea beets and sugar beets. Sea beet (*B. v. maritima*) accessions are divided into two groups based on geographic provenance, i.e. Atl: Atlantic region; Med: Mediterranean area.

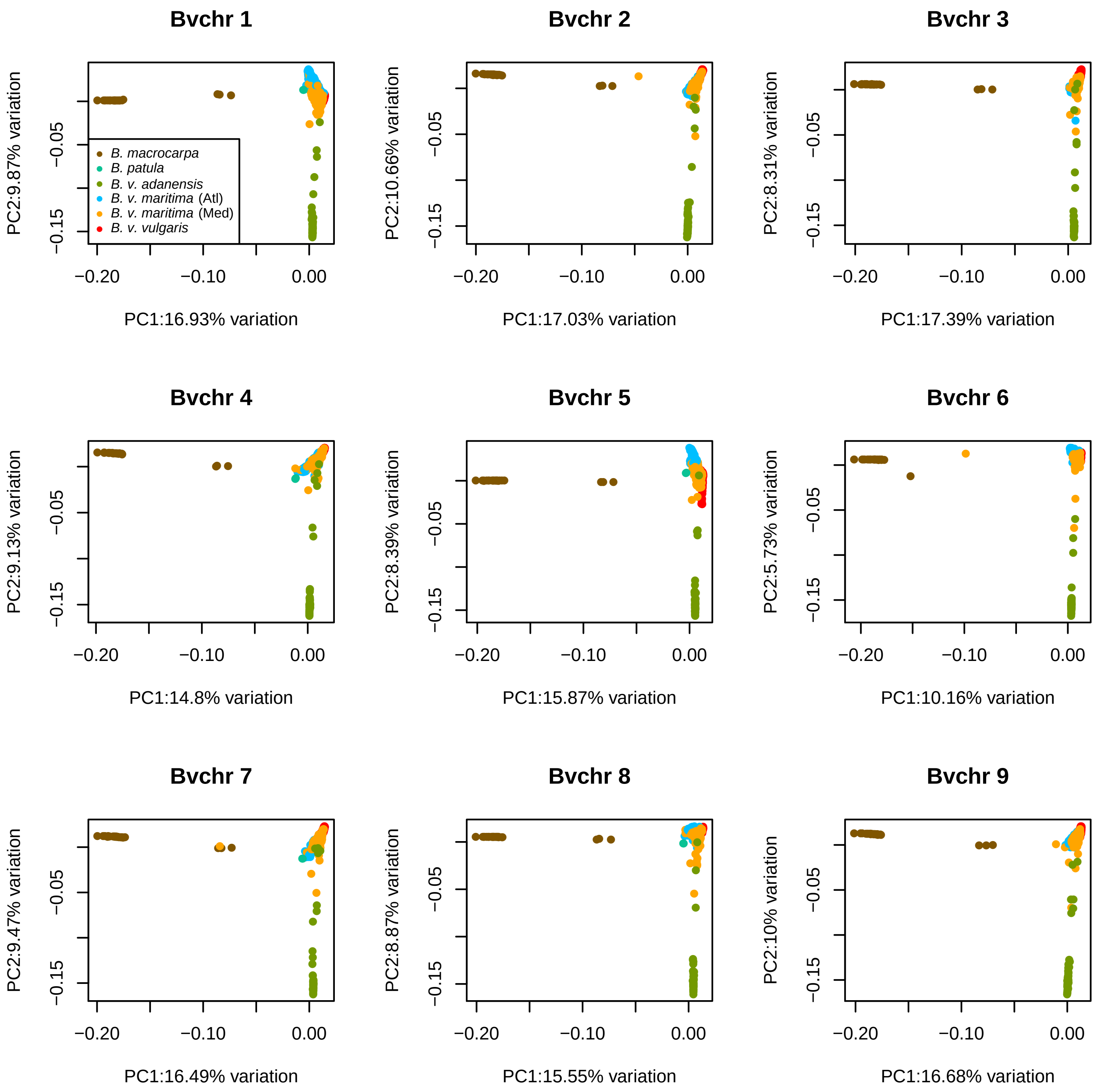

Figure S5: Principal component analysis (PCA) for the nine beet chromosomes separately, based on variants in the genus *Beta*. Sea beet (*B. v. maritima*) accessions are divided into two groups based on geographic provenance, i.e. Atl: Atlantic region; Med: Mediterranean area.

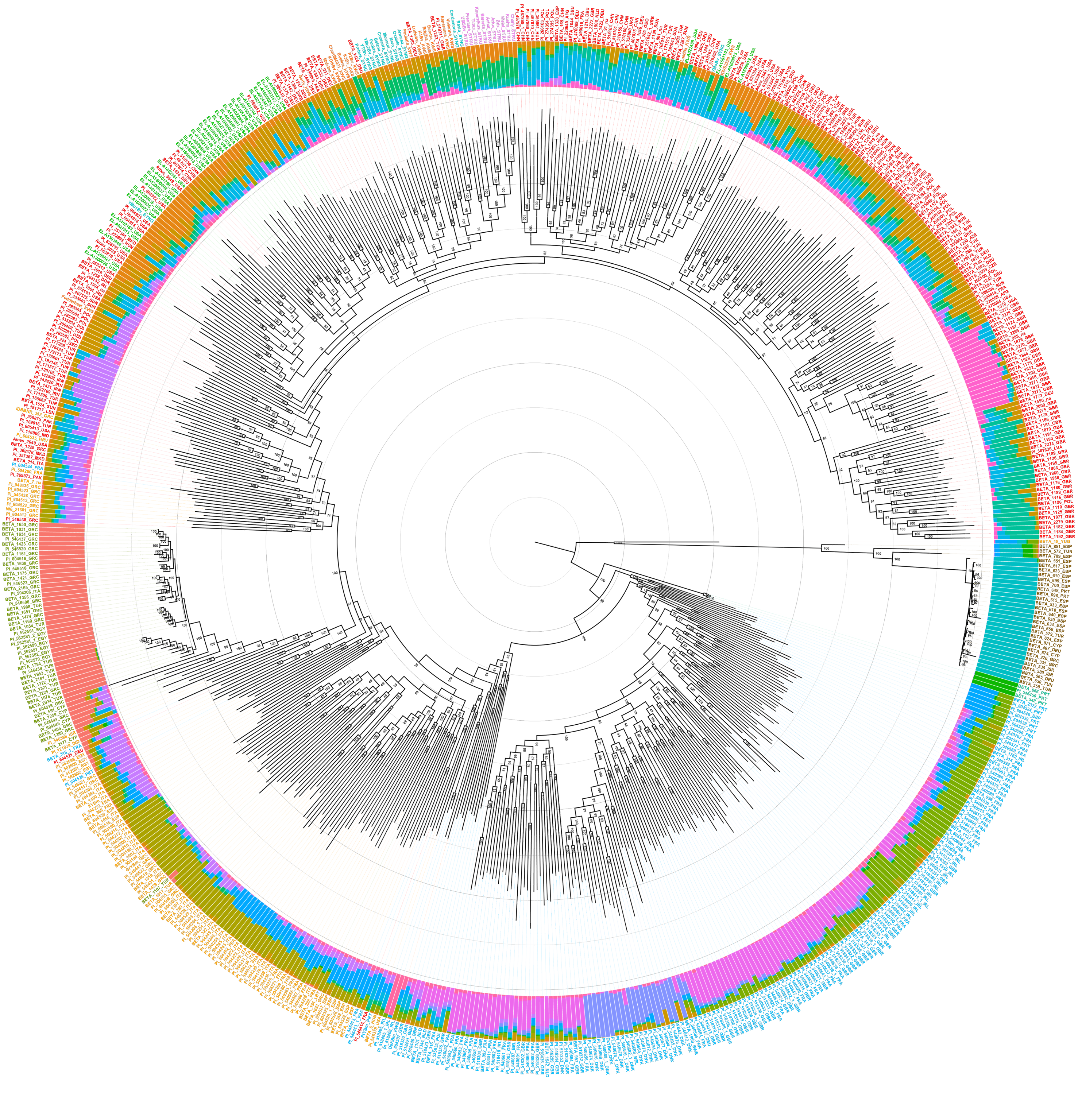

Figure S6: Maximum likelihood tree calculated based on 4DTv sites detected in *Beta* accessions. Ancestry proportions from an admixture analysis are annotated onto the tree.

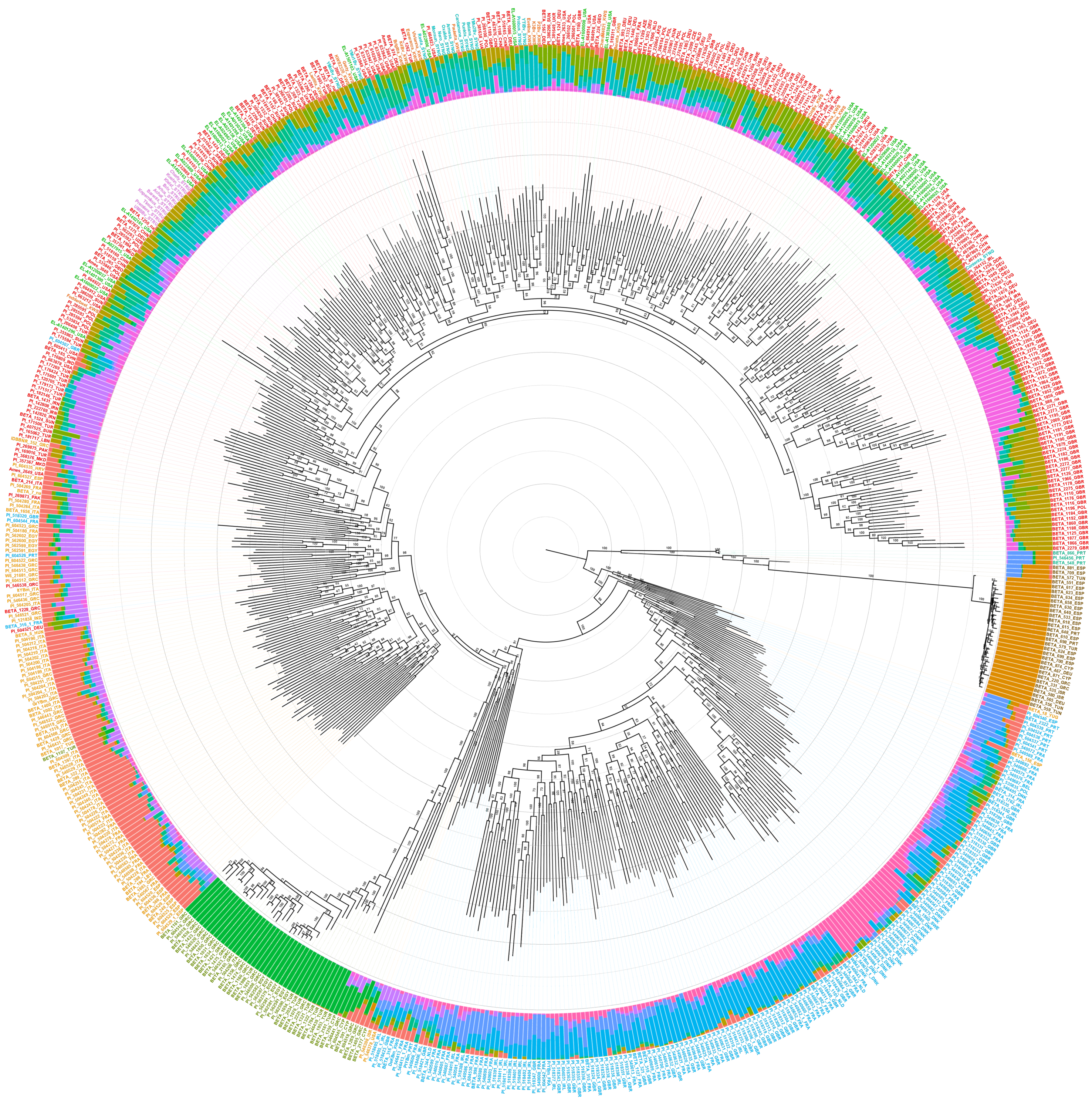

Figure S7: Maximum likelihood tree calculated based on intergenic variant sites detected in *Beta* accessions. Ancestry proportions from an admixture analysis are annotated onto the tree.

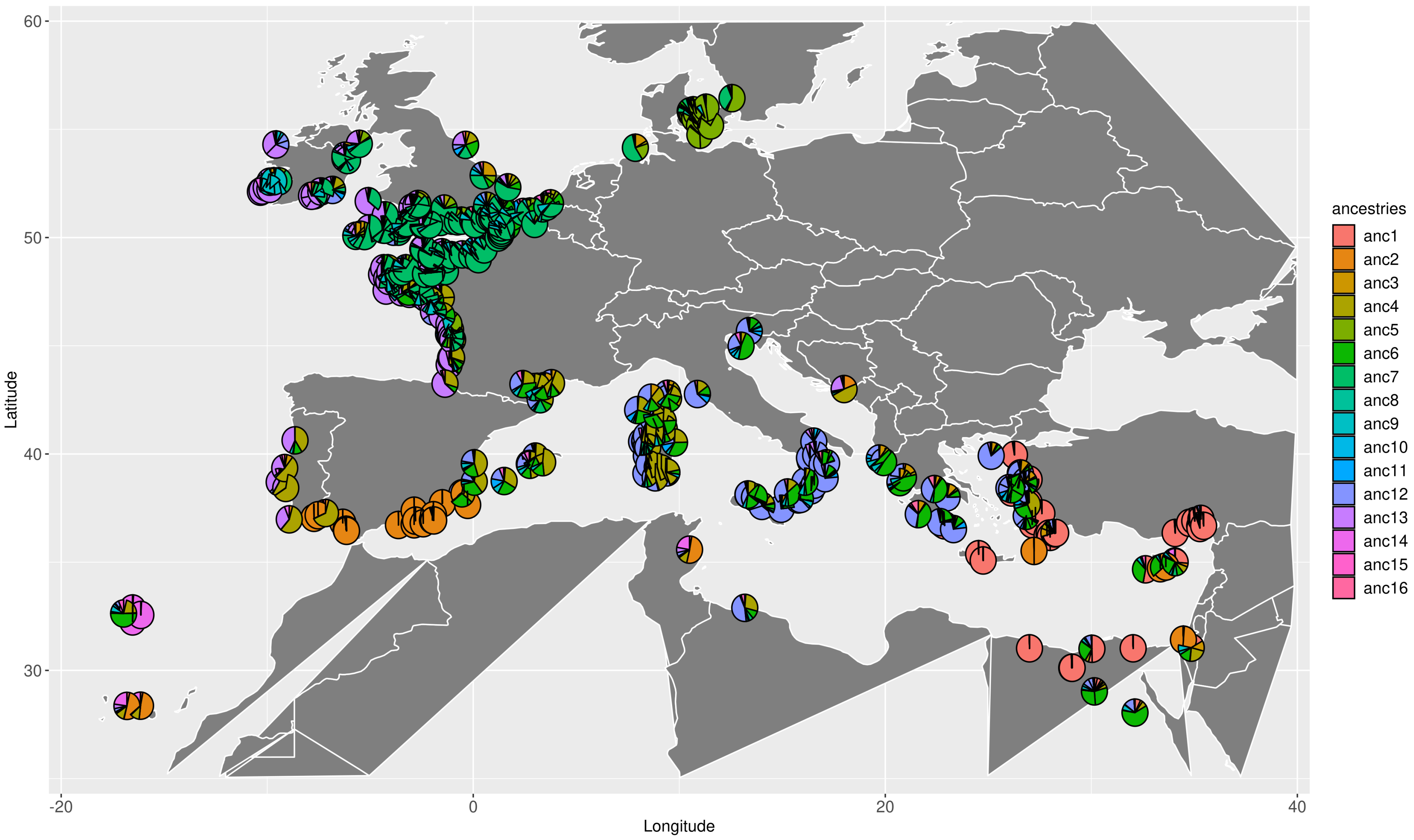

Figure S8: Admixture pie charts of wild beet species based on exonic variants (k=16).

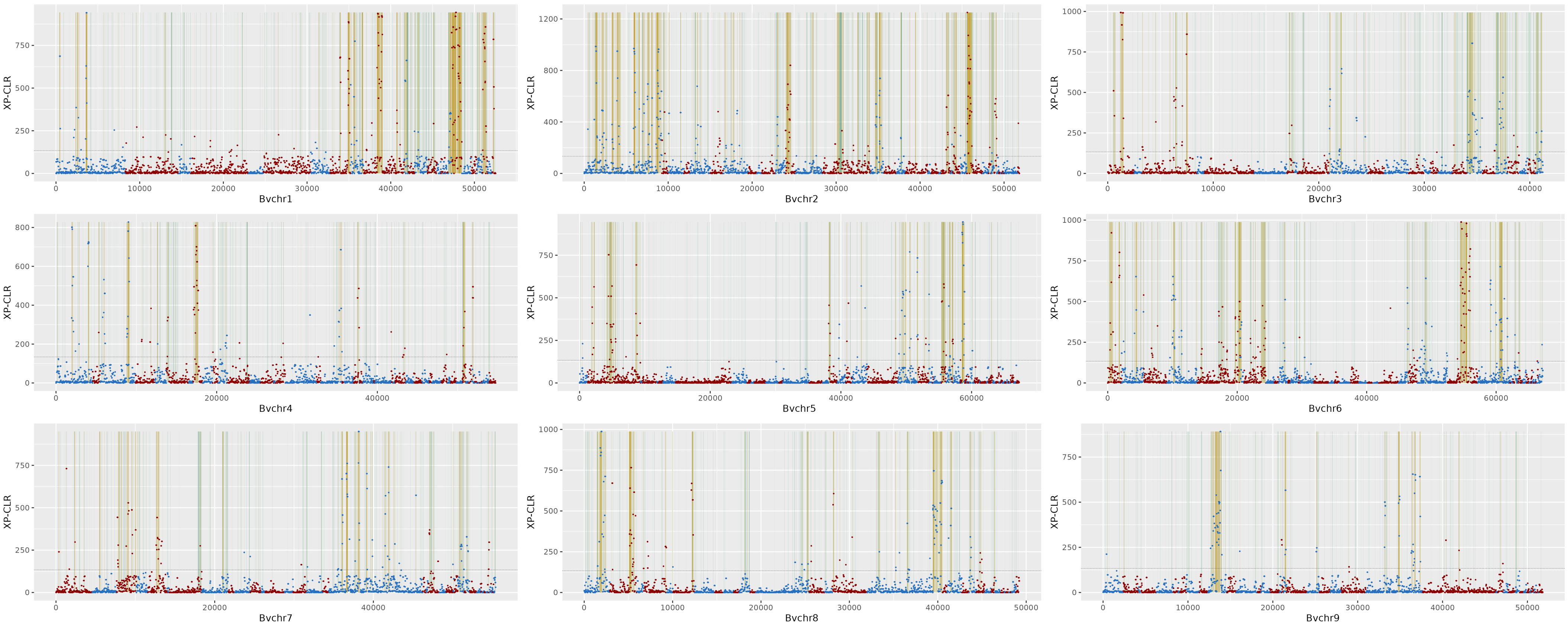

Figure S9: Variation-poor regions in sugar beet using a sliding window approach and a cross-population composite likelihood ratio test (XP-CLR). Green vertical bars indicate low variation in 1 kbp windows, red and blue data points (alternating by scaffold) represent XP-CLR values along RefBeet-1.2 (tics in kbp), regions of high XP-CLR values are highlighted in yellow. The horizontal line marks the 95%ile of XP-CLR values.

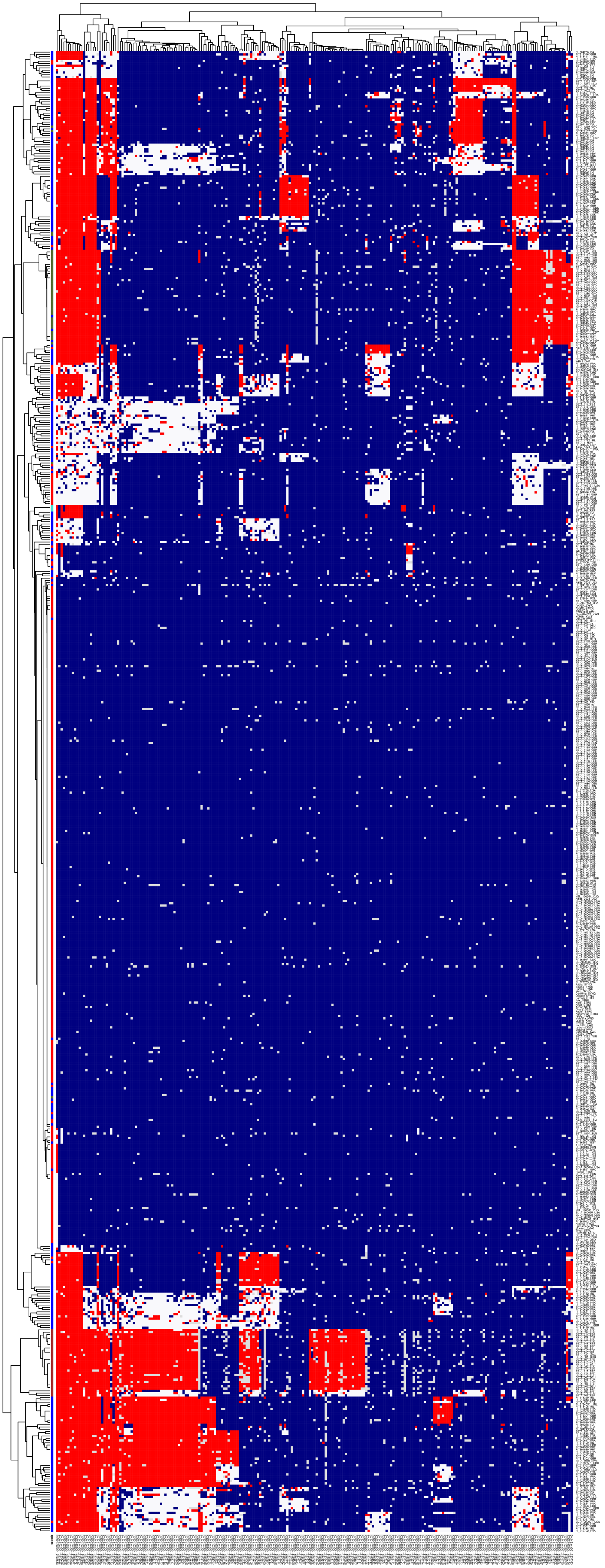

Figure S10: Genotypes of all beet accessions used in this study for the locus encoding Bv2_025460_xhyw.t1, a gene involved in UVR-B radiation response, and top candidate according to GO term enrichment analysis of genes located in sugarbeet variation deserts. Rows correspond to accessions (names on the right), columns are positions as indicated at the bottom; colors on the left (thin vertical bar) distinguish accessions by taxonomic affiliation (cyan: *B. patula*; blue: *B. v. maritima*; red: *B. v. vulgaris* (sugar beet); olive: *B. v. adanensis*; maroon: *B. macrocarpa*). Colors in the plot area represent the variation status per accession per position, whereby homozygous reference = blue; heterozygous = white, homozygous variant = red, missing values = grey. While several haplotypes exist in wild beets (top and bottom area of the plot) there is almost exclusively only one haplotype in sugar beets (large blue area).

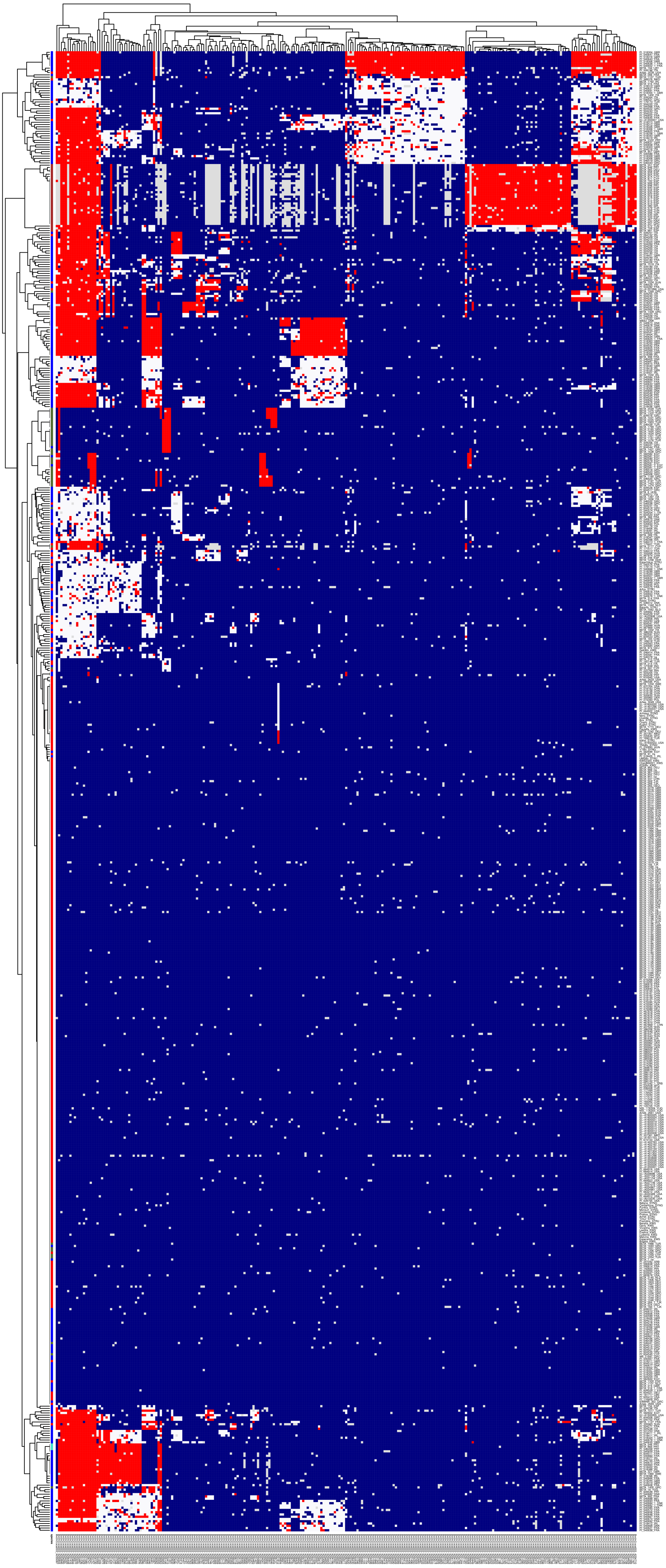

Figure S11: Genotypes of all beet accessions used in this study for the locus encoding Bv3_054860_wjth, a gene involved in inositol transport, and top candidate according to GO term enrichment analysis of genes located in sugarbeet variation deserts. Details as in Figure S10. While several haplotypes exist in wild beets (top and bottom area of the plot) there is almost exclusively only one haplotype in sugar beets (large blue area).

Supplementary Tables

Table S1: Accessions analysed in this study. Column designations: Accession name and origin: Accession identifier and country or source of origin; "IDBBNR_352" is listed under the accession number "1989" in the Greek Genebank (IDBBNR numbers are not maintained anymore). Reference: Source of sequencing data. Source: Database or accession provider. SeqID: Internal sequencing data identifier. Species: Taxonomic classification as provided by passport data or as proposed by Wascher et. al. (2022). Number of reads: Raw sequencing reads. Number of quality filtered reads: Sequencing reads after quality filtering.

Table S2: Positions of windows along RefBeet-1.2 that were identified as private sugar beet variation deserts. Column 1: Scaffold name. Column 2: Start position of window. Column 3: End position of window. Column 4: Windows that had a low number of variants and additionally high XP-CLR values are marked with "2", otherwise marked with "1".

Table S3: Significantly enriched gene ontology (GO) terms of category "biological process" for genes that are (partially) located in private sugar beet variation deserts. Column designations: ID: GO term identifier; Description: GO term description; GeneRatio: ratio of input genes that are annotated in a GO term; BgRatio: ratio of all genes that are annotated in this GO term; pvalue: p-value; qvalue: q-value; geneID: BeetSet-2 identifier of genes associated with this GO term; Count: number of input genes associated with this GO term.
